# A panel of tumor markers, calreticulin, annexin A2, and annexin A3 in upper tract urothelial carcinoma identified by proteomic and immunological analysis

**DOI:** 10.1186/1471-2407-14-363

**Published:** 2014-05-23

**Authors:** Chih-Ming Lu, Jen-Jie Lin, Han-Hsiang Huang, Ying-Chin Ko, Jue-Liang Hsu, Jiing-Chuan Chen, Zhong-Hao Din, Yu-Jen Wu

**Affiliations:** 1 Department of Urology, Dalin Tzu Chi Hospital, Buddhist Tzu Chi Medical Foundation, Chiayi, Taiwan; 2 Graduate Institute of Veterinary Medicine, National Pingtung University of Science and Technology, Pingtung, Taiwan; 3 Department of Beauty Science, Meiho University, Pingtung, Taiwan; 4 Institute of Medicine, China Medical University, Taichung, Taiwan; 5 Graduate Institute of Biotechnology, National Pingtung University of Science and Technology, Pingtung, Taiwan; 6 Department of Food Science and Nutrition, Meiho University, Pingtung, Taiwan; 7 Graduate Institute of Applied Healthy and Biotechnology, Meiho University, Pingtung, Taiwan

**Keywords:** Upper tract urothelial carcinoma, Proteomic, Urine, Annexin A2, Annexin A3, Calreticulin, Zn-alpha-2-glycoprotein

## Abstract

**Background:**

Upper tract urothelial carcinoma (UTUC) is a tumor with sizable metastases and local recurrence. It has a worse prognosis than bladder cancer. This study was designed to investigate the urinary potential tumor markers of UTUC.

**Methods:**

Between January 2008 and January 2009, urine was sampled from 13 patients with UTUC and 20 healthy adults. The current study identified biomarkers for UTUC using non-fixed volume stepwise weak anion exchange chromatography for fractionation of urine protein prior to two-dimensional gel electrophoresis.

**Results:**

Fifty five differential proteins have been determined by comparing with the 2-DE maps of the urine of UTUC patients and those of healthy people. Western blotting analysis and immunohistochemistry of tumor tissues and normal tissues from patients with UTUC were carried out to further verify five possible UTUC biomarkers, including zinc-alpha-2-glycoprotein, calreticulin, annexin A2, annexin A3 and haptoglobin. The data of western blot and immunohistochemical analysis are consistent with the 2-DE data. Combined the experimental data in the urine and in tumor tissues collected from patients with UTUC, the crucial over-expressed proteins are calreticulin, annexin A2, and annexin A3.

**Conclusions:**

Calreticulin, annexin A2, and annexin A3 are very likely a panel of biomarkers with potential value for UTUC diagnosis.

## Background

Urothelial (transitional cell) carcinoma is the most common malignancy in epithelium of the bladder, ureter, and kidney. Upper tract urothelial carcinoma (UTUC) is relative rare and accounting for 5% of all urothelial neoplasms. Three quarters of the UTUC incidence occur in renal pelvis. The overall incidence rate of UTUC in Taiwan is higher than that in worldwide else [1]. Investigation suggested this is due to a wide usage of herb drug such as Aristolochia manshuriensis Kom and analgesics in Taiwan. Bladder cancer rarely migrates to upper tract. Patients with UTUC have a higher risk of developing bladder cancers (30-50%) [2]. In contrast to the application of transurethral resection and intravesical chemotherapy to avoid cystectomy in life time for early stage bladder cancer, UTUC mostly undergo radical nephroureterectomy with the risk of hemodialysis. Oftentimes UTUC has a poorer prognosis than bladder cancer [3,4]. Reports regarding medically useful tumor markers for UTUC are very limited. The common manifestations of urothelial carcinomas are gross hematuria. Most of the urological diseases, such as urinary tract infection, benign prostatic hyperplasia, and urinary calculi may also show hematuria. The available diagnostic methods for UTUC are urine cytology, CT urography, and cystoscopy. However, the sensitivity and specificity of these manners are not satisfactory.

Proteomic analysis of tissues or body fluids has been applied in clinical detections. Identification of disease biomarkers is potentially useful for diagnosis, monitoring disease progress as well as evaluation of therapies. Two-dimensional gel electrophoresis (2-DE) is an essential tool for proteome studies. It has been used in biomarker identification for diagnosis and clinical monitoring of diseases [5]. Urine contain large amount of high-abundance proteins such as immunoglobulin heavy and light chain proteins, generally obscure low-abundance proteins on 2-DE maps. Previously a non-fixed volume stepwise isocratic elution weak anion exchange (WAX) chromatography was used to fractionate healthy urine proteins into four groups prior to performing two dimensional electrophoresis and the results indicated that low-abundance proteins can be detected on 2-DE maps and identified by LC-MS/MS analysis after the removal of high-abundance proteins [6]. Herein our study identifies biomarkers for UTUC using non-fixed volume stepwise WAX chromatography for fractionation of urine protein prior to 2-DE and using comparative proteomic analysis for the detection of differential proteins. The urine proteins of patients with UTUC were fractionated into four fractions following the same procedures for that of healthy people. Differential protein spots were detected by comparing the 2-DE maps of UTUC patients with those of healthy people. The differential protein spots were identified and further evaluated by urine western blotting analysis while tumor tissues collected from UTUC patients were further assessed by immnohistochemistry and western blot to provide potentially important protein markers for UTUC.

## Methods

This protocol was approved by the Institute Review Board of the Buddhist Dalin Tzu Chi Hospital (Approval No. B09601020). We obtained all patients’ consent for both the urine collection and the tumor sample collection and use.

### Collection of urine of healthy people

Urine samples were collected from 20 healthy individuals including eleven males and nine females, aged between 18 and 54 years, who had neither history of drug administration nor renal disorders during sample collection period. All females had no menstruation at the time of sample collection. A 100 ml urine sample was collected for every person in the mornings and the samples were combined together. The urine samples were treated with protease inhibitor cocktail to avoid proteolysis. They were then centrifuged at 12,000 rpm to remove insoluble material and cell debris at 4°C. Stirred Ultrafiltration Cell 8400 (Millipore, Billerica, MA, USA) and YM5 membrane (5000 molecular weight cut-off) were used to concentrate the solution and remove small interference molecules. The concentrated urine samples had a final volume of 50 ml and were stored at -80°C for future use.

### Collection of urine of patients with UTUC

The incidence of UTUC is relatively rare and it is difficult to have a large number of patients in a local area. A total of 13 patients with UTUC enrolled in this study between January, 2008 and January, 2009. There were 5 male and 8 female. The average age was 62.6 years (33–80 years old). All the patients were newly diagnosed and confirmed by histological examination. A 100 ml urine sample was collected for each patient prior to surgery and the samples were combined together. The further treatments of the samples adopted the same procedures for the samples of healthy people.

### Collection of tumor tissue of patient with UTUC

The tissue specimen were harvested by nephroureterectomy for UTUC. In addition, the normal control epithelium was resected from approximately 1 cm away from the tumor. The tissue was collected at least 5 mm × 5 mm × 5 mm in size. These tissues were immediately stored in liquid nitrogen for later analysis.

### Fractionation of urine proteome by non-fixed volume stepwise WAX

According to the previous procedures [6], a column (5 cm × 10 cm) packed with 50 gram DEAE-Sephacel (GE Healthcare) which was equilibrated with 50 mM Tris–HCl buffer for five times before use. Twenty ml of concentrated urine samples were dialyzed at 4°C overnight and then loaded to the column. First the column was eluted by 50 mM Tris–HCl buffer without salt at a flow rate of 40 ml/hr. The column was eluted until no proteins were detected in the eluent by Bradford dye assay. A total combined eluent was collected and concentrated by Stirred Ultrafiltration Cell 8400 and YM5 membrane to a volume of 50 ml. The sample was called fraction unbound. Then, a solution of 50 mM NaCl/50 mM Tris–HCl buffer was used to elute the column until no protein was detected in the eluent and a total solution was collected and concentrated by the same procedure to obtain the fraction NaCl-1. 100 mM NaCl/50 mM Tris–HCl buffer was collected following the same procedures to obtain fraction NaCl-2. 1 M NaCl/50 mM Tris–HCl buffer was collected for the last elution to obtain fraction NaCl-3.

### Urine proteins precipitation and two-dimensional gel electrophoresis

The urine protein mixture in the supernatant was precipitated out overnight at -20°C by 100 ml 10% TCA/Acetone solution containing 20 mM DTT [7]. The pellet was rinsed in cold acetone containing 20 mM DTT and dried by SpeedVac, then resuspended in a rehydration buffer (8 M urea, 0.5% CHAPS, 0.5% IPG buffer, 20 mM DTT, 0.002% bromophenol blue) at 4°C overnight. The protein contents were determined using 2-D Quant Kit (GE Healthcare).

The first dimension electrophoresis (isoelectric focusing) was performed GE Healthcare Ettan IPGphor 3 using the reported procedure [8]. Urine proteins (50 μg) were loaded on 11-cm strip. Every 11-cm IPG strip (*pI* 4–7 and *pI* 3-10NL, Immobiline DryStrip) was rehydrated at 50 V for 12 h, then focused according to the preset program: 200 V (2 h), 500 V (1 h), 1,000 V (1 h), 4,000 V (2 h), 8,000 V (3 h), until the total Vh reached 32,060. Then second dimension electrophoresis was done 15% SDS-PAGE run at 150 V for 7 h. The second dimension electrophoresis was used SE 600 Ruby electrophoretic unit (Hoeffer). The 2-DE gels were stained with silver staining and then subjected to image analysis with the PDQuest 2-D software (version 7.1.1). 2-DE images were taken in triplicate for each sample and normalized prior to statistical analysis.

### Protein spot identification by LC-MS/MS

The protein spots of interest were excised, destained and then subjected to tryptic in-gel digestion as described in a previous report [9].

The peptide solution was concentrated for the following LC-MS/MS analysis. After desalting with a Millipore ZIP plate (Millipore), the above peptide mixture was separated by nanoflow reversed phase C18 chromatography on nano LC using the Agilent NanoLC 1200 System and Agilent Zobax 2.1 mm × 150 mm C18 column. MS analysis was performed using a AB SCIEX QTRAP® 5500Q mass spectrometer (Applied Biosystems, CA, USA). The scan range was from m/z 100 to 1000 for MS. The raw data was processed into a text file format of WIFF with Analyst 1.5.1. MASCOT was used in searching for protein identification by NCBInr protein database.

### Western blot analysis

Western blot was conducted to verify the regulation of 5 differential 2-DE-detected proteins. The protein samples under reducing conditions were loading 20 μg total proteins and separated by 12.5% SDS-PAGE. The proteins on the gel were then transferred to PVDF membranes. The membranes were incubated with rabbit antibodies against human calreticulin (CALR), annexin A2, annexin A3, haptoglobin (Hp), and zn-alpha-2-glycoprotein (ZAG) at 4°C for 2 h or overnight. The membranes were washed three times in PBST (10 mM NaH_2_PO_4_, 130 mM NaCl, 0.05% Tween 20), then incubated with the second antibodies (goat anti-rabbit with horseradish peroxidase conjugated, 1:5,000 in blocking solution) for 1 h. After washing with PBST for three times, the blots were visualized through chemiluminesence by adding ECL western blotting reagents.

### Immunohistochemistry

Using the Bond-Max autostainer (Leica Microsystems), slides were stained with annexin A2 polyclonal antibodies (1:50; ProteinTech Group, Chicago, IL, USA) applied at room temperature for 1 h. Briefly, formalin-fixed and paraffin-embedded tissue array specimens were in Tris–HCl buffer (50 mM Tris, 130 mM NaCl, 0.05% Tween 20), rehydrated through serial dilutions of alcohol, and washed in PBST. Slides were stained with previously mentioned antibodies was performed on the fully automated Bond-Max system using onboard heat induced antigen retrieval and a Leica Refine polymer detection system (Leica Microsystems). Diaminobenzidine was used as the chromogen (Leica Microsystems) in all these immunostainings. Negative controls were obtained by excluding the primary antibody. Appropriate positive controls were used throughout the study. These slides were mounted with gum for examination and the images were captured by the Olympus BX51 microscopic/DP71 Digital Camera System (Ina-shi, Nagano, Japan) for study comparison.

## Results

### The characteristics of UTUC patients and controls

There were eight males and five females in the UTUC group while eleven males and nine females are in the healthy control group. The mean ages of UTUC patients and healthy controls were 62.6 years (range 33–80 years) and 34.2 years (range 18–54 years), respectively. In the UTUC group, there were 8 (61.5%) patients with stage pT1/T2, 5 (38.5%) with stage pT3. Among these UTUC patients, 23.1% (3/13) had grade 3 tumors. Most of the patients had solitary tumors (69.3%) and big lesions (76.9% with >4 cm in diameter). However, only 66.7% (6/9) patients showed carcinoma cell in urine cytology (Table 1).

**Table 1:** The characteristics of UTUC patients and controls

	**UTUC**	**Control**
	N = 13	N = 20
Sex		
Male	8 (61.5%)	11 (55.0%)
Female	5 (38.5%)	9 (45.0%)
Mean age (years)	62.6 (33–80)	34.2 (18–54)
Tumor grade		
G1	7 (53.8%)	-
G2	3 (23.1%)	-
G3	3 (23.1%)	
T Stage		
T1	4 (30.7%)	-
T2	4 (30.7%)	-
T3	5 (38.5%)	-
Urine cytology		
Positive	6 (46.2%)	-
Negative	3 (23.1%)	-
Unknown	4 (30.7%)	-
Tumor size		
<1 cm	0 (0%)	-
1-4 cm	3 (23.1%)	-
>4 cm	10 (76.9%)	-
Tumor multiplicity		
Solitary	9 (69.3%)	-
Multiple	4 (30.7%)	-

### 2-DE maps of urine proteome of healthy people and UTUC patients

**Figure 1:**
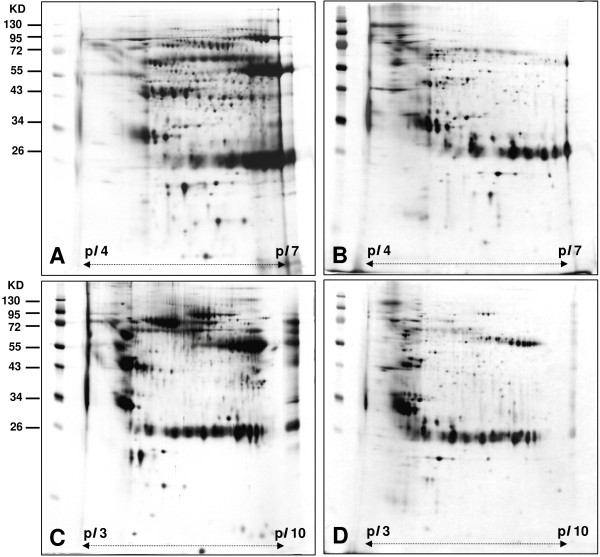
**Two-DE maps of urine proteome of UTUC and healthy people. (A)(C)** 2-DE maps were for UTUC and **(B)(D)** for healthy people.

A 100 ml urine sample was collected from each of 20 healthy people and 13 UTUC patients. In order to eliminate the individual difference, the samples were combined together. The proteins were precipitated by 10% TCA/Acetone after dialysis. An equal amount of 50 g per gel was resolved in 2-DE using IPG strip (*pI* 4*–*7 and *pI* 3-10NL). The protein spots were visualized with silver stain. The differential proteins in the urine proteome of UTUC patients were examined by comparing with those of healthy people (Figure 1).

### Volume stepwise WAX with NaCl solutions

**Figure 2:**
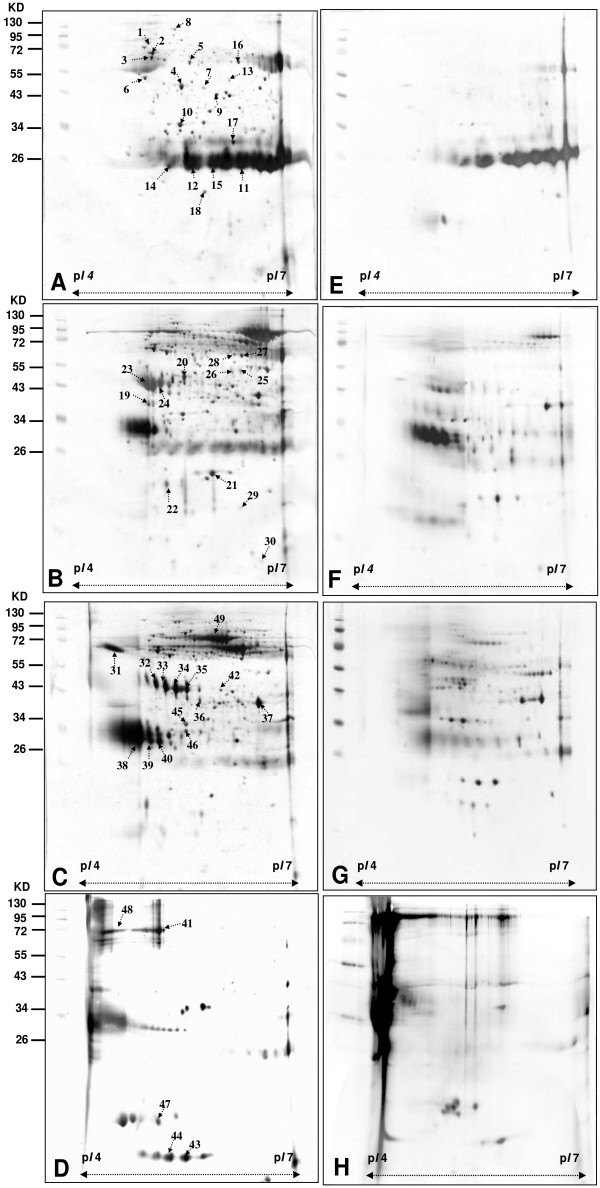
**2-DE maps of fractions of urine proteome of UTUC patients and healthy people obtained by non-fixed volume stepwise elution DEAE-Sephacel anion exchange chromatography.** (*pI* 4–7) **(A)**, **(E)** unbound Proteins in fraction **(B)**, **(F)** Proteins in fraction NaCl-1 obtained by elution with 50 mM NaCl. **(C)**, **(G)** Proteins in fraction NaCl-2 obtained by elution with 100 mM NaCl. **(D)**, **(F)** Proteins in fraction NaCl-3 obtained by elution with 1 M NaCl. Images **A**, **B**, **C** and **D** are for UTUC patients. Images **E**, **F**, **G** and **H** for healthy people.

**Figure 3:**
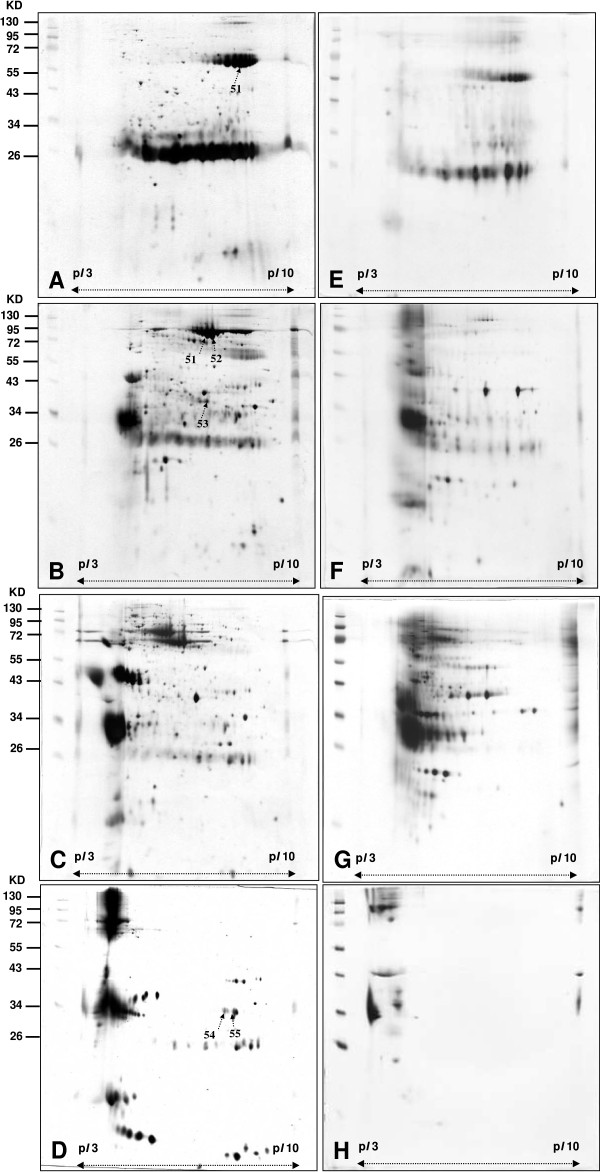
**2-DE maps of fractions of urine proteome of UTUC patients and healthy people with obtained by non-fixed volume stepwise elution DEAE-Sephacel anion exchange chromatography.** (*pI* 3–10 NL) **(A)**, **(E)** unbound Proteins in fraction **(B)**, **(F)** Proteins in fraction NaCl-1 obtained by elution with 50 mM NaCl. **(C)**, **(G)** Proteins in fraction NaCl-2 obtained by elution with 100 mM NaCl. **(D)**, **(F)** Proteins in fraction NaCl-3 obtained by elution with 1 M NaCl. Images **A**, **B**, **C** and **D** are for UTUC patients. Images **E**, **F**, **G** and **H** for healthy people.

A direct 2-DE analysis of a complex protein sample would encounter resolution problem. Usually not all proteins can be identified on a 2-DE map. In order to identify the differential proteins in the urine proteome of patients with UTUC in comparison with that of healthy people, the urine proteome of patients with UTUC was also fractionated into four fractions using the same experimental procedures and conditions. The 2-DE maps of the four fractions of patient with UTUC were shown in Figure 2 (2A for the fraction Unbound, 2B for the fraction NaCl-1, 2C for the fraction NaCl-2, and 2-DE for the fraction NaCl-3; 2A-2D for UTUC patient, 2E-2H for healthy people). The Figure 3A-3F showed the corresponding 2-DE maps of the four fractions of urine proteome of UTUC patients and healthy people run on *pI* 3–10 NL. (3A-3D for UTUC patient, 3E-3H for healthy people)

A side-by-side comparison between the 2-DE maps of each fraction of patients and those of healthy people showed clearly different staining patterns. A total of 208 protein spots were detected in Figure 2A comparing with 53 protein spots in Figure 2E, a total of 369 spots were detected in Figure 2B comparing with 102 protein spots in Figure 2F, a total of 350 spots were detected in Figure 2C comparing with 143 protein spots for Figure 2G, and a total of 101 spots were detected in Figure 2D comparing with 31 protein spots in Figure 2H by comparative analysis using PDQuest 2-D software (version 7.1.1). A total of 1028 protein spots were shown in Figure 2A-2D comparing with a total of 329 protein spots in Figure 2E-2H.

Analysis of all the differential protein spots would encounter practical difficulties. Identification of fifty-five (spot 1 ~ 55) differential protein spots by LC-MS/MS after excision and in-gel digestion was carried out and the identities are represented in Table 2. The differential proteins spots were defined as the proteins only present in patients or showed intensity difference between patients and healthy people using PDQuest 2-D software. The differential proteins include zinc-alpha-2-glycoprotein (ZAG), heparan sulfate proteoglycan (HSPG), alpha-1-microglobulin/bikunin precursor, calreticulin (CALR), haptoglobin (Hp), serotransferrin, ATP-binding cassette sub-family A member 9, annexin A2, annexin A3 sorting nexin-14, Serum albumin, spermatogenesis-associated protein 7, DNA replication licensing factor MCM2, proline-serine-threonine phosphatase-interacting protein 1, dermcidin precursor, glial fibrillary acidic protein, syntaxin-binding protein 4, and transthyretin.

**Table 2:** Summary of differential protein spots of urine between patients with UTUC and healthy people identified by LC-MS/MS

**Spot no**	**Protein name**	**Accession no**	**Calculate Mw/*pI***	**Peptide matched**	**Sequence covered%**	**MASCOT score**
1	ATP-binding cassette sub-family A member 9	Q8IUA7	184.2/6.49	10	1	38
2	sorting nexin-14	Q9Y5W7	102.8/6.4	17	3	34
3	serum albumin precursor	P02768	69.3/5.92	1	2	57
4	F-box/WD repeat protein 1A	Q9Y297	68.8/8.3	29	3	33
5	spermatogenesis-associated protein 7	Q9P0W8	67.6/5.9	3	4	34
6	actin	P62736	41.9/5.23	2	9	45
7	cytokeratin 1	P04264	65.9/8.16	3	3	40
8	DNA replication licensing factor MCM2	P49736	101.8/5.34	40	1	50
9	proline-serine-threonine phosphatase-interacting protein 1	O43586	47.5/5.53	6	4	40
10	X box-binding protein 1	P17861	28.6/9.71	18	3	35
11	immunoglobulin kappa chain C region	P01834	11.6/5.58	16	49	151
12	immunoglobulin kappa chain V-I region AG	P01593	11.9/5.67	4	31	176
13	hemopexin precursor	P02790	51.6/6.55	4	7	86
14	prostaglandin-H2 D-isomerase precursor	P41222	21.0/7.66	3	12	33
15	immunoglobulin kappa chain C region	P01834	11.6/5.58	22	49	217
16	gelsolin precursor	P06396	85.6/5.9	9	7	208
17	SH3-containing GRB2-like protein 3	Q99963	39.2/5.27	16	8	32
18	hemoglobin subunit beta	P68871	15.9/6.75	9	32	214
19	inter-alpha-trypsin inhibitor heavy chain H4	Q14624	103.2/6.51	13	10	243
20	cytokeratin 10	P13645	59.4/5.13	2	4	38
21	basement membrane-specific heparan sulfate proteoglycan core protein precursor	P98120	468.8/6.06	116	4	2114
22	retinol Binding Protein 4	P02753	22.9/5.76	45	35	233
23	Zn-alpha2-glycoprotein	P25311	34.7/5.71	14	45	378
24	Zn-alpha2-glycoprotein	P25311	34.7/5.71	5	16	98
25	fibrinogen gamma chain precursor	P02679	51.4/5.37	19	24	201
26	fibrinogen gamma chain precursor	P02679	51.4/5.37	5	11	38
27	serotransferrin precursor	P02787	77.0/6.81	7	5	48
28	serotransferrin precursor	P02787	77.0/6.81	18	13	88
29	annexin A3	P12429	36.3/5.63	3	4	37
30	dermcidin precursor	P81605	11.2/6.08	2	16	48
31	calreticulin	P27797	48.1/4.29	19	36	493
32	haptoglobin	P00738	45.1/6.13	39	13	432
33	haptoglobin	P00738	45.1/6.13	41	20	466
34	haptoglobin	P00738	45.1/6.13	49	17	668
35	haptoglobin	P00738	45.1/6.13	19	15	271
36	haptoglobin	P00738	45.1/6.13	5	13	77
37	glial fibrillary acidic protein	P14136	49.8/5.42	2	4	43
38	alpha-1-microglobulin/bikunin precursor	P02760	38.9/5.95	41	14	698
39	alpha-1-microglobulin/bikunin precursor	P02760	38.9/5.95	48	15	732
**Spot no**	**Protein name**	**Accession no**	**Calculate Mw/*pI***	**Peptide matched**	**Sequence covered%**	**MASCOT score**
40	alpha-1-microglobulin/bikunin precursor	P02760	38.9/5.95	45	23	667
41	syntaxin-binding protein 4	Q6ZWJ1	61.7/5.16	4	7	40
42	mRNA cap guanine-N7 methyltransferase	O43148	54.8/6.3	11	3	52
43	transthyretin precursor	P02766	15.8/5.52	13	48	217
44	transthyretin precursor	P02766	15.8/5.52	8	40	142
45	alpha-1-microglobulin/bikunin precursor	P02760	38.9/5.95	5	11	63
46	alpha-1-microglobulin/bikunin precursor	P02760	38.9/5.95	13	11	198
47	transthyretin precursor	P02766	15.8/5.52	4	14	46
48	serine/threonine-protein kinase MRCK beta	Q9Y5S2	194.1/5.91	3	2	56
49	serum albumin precursor	P02768	69.3/5.92	51	25	594
50	serotransferrin precursor	P02787	77.0/6.81	42	21	441
51	serum albumin precursor	P02768	69.3/5.92	92	26	1359
52	serum albumin precursor	P02768	69.3/5.92	48	26	662
53	annexin A2	P07355	38.58/7.57	28	37	505
54	Ig gamma-1 chain C region	P01857	36.0/8.46	20	26	190
55	Ig gamma-1 chain C region	P01857	36.0/8.46	12	25	104

### Western blot analysis of urine and tissue

**Figure 4:**
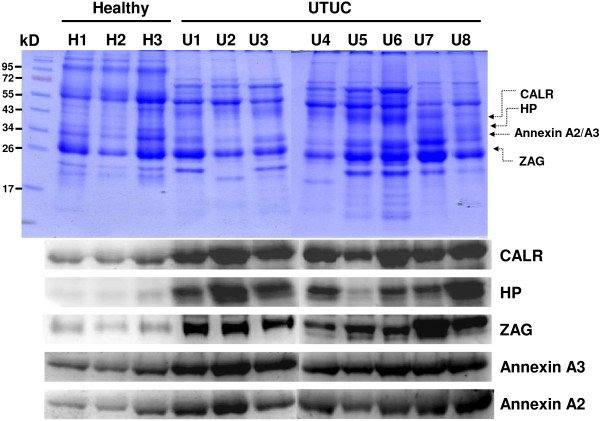
**Comparison of CBR staining of western blot analysis of zinc-alpha-2-glycoprotein (ZAG), Calreticulin (CALR), Annexin A2, Annexin A3 and hapatoglobin (Hp) in urine of healthy people and urine of UTUC patients, each with a loading of 20 μg urine protein.** Lane H1-H3 for individual urine of three healthy people and Lane U1-U5 for individual urine of five UTUC patients.

**Figure 5:**
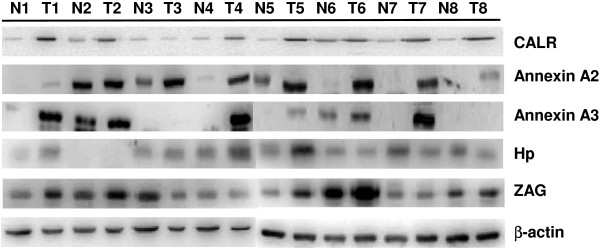
**Side-by-side comparison using western blot analysis of zinc-alpha-2-glycoprotein (ZAG), Calreticulin (CALR), annexin A2, annexin A3 and hapatoglobin (Hp) of tumor tissues and normal tissuess, each well with a loading of 20 μg protein.** Tumor tissue was presented by T and normal tissue was presented by N.

Western blot analysis of specific urine proteins was performed to verify the 2-DE results. Western blot data of ZAG, CALR, annexin A2, annexin A3 and Hp in the urine of both patients and healthy people are shown in Figure 4. The results indicated that clearly stronger expression of ZAG, CALR, annexin A2, annexin A3 and Hp in the urine of patients in comparison with the counterparts in the urine of healthy people. This confirmed the 2-DE data of ZAG (spots 23,24), CALR (spot 31), annexin A2 (spot 53), annexin A3 (spot 29) and Hp (spot 32 ~ 36). Side-by-side comparisons of CALR, ZAG, annexin A2, annexin A3 and Hp showed the different expression between tumor tissues and normal tissues. The expression of CALR, annexin A2, and annexin A3 in the tumor tissues was higher than that in the normal tissues. These results are in accordance with the western blot data on the urine samples of UTUC patients as CALR, annexin A2, and annexin A3 are the reasonable connection between urine proteins of UTUC and the tumor tissues (Figure 5).

### Immunoreactivity of annexin A2

**Figure 6:**
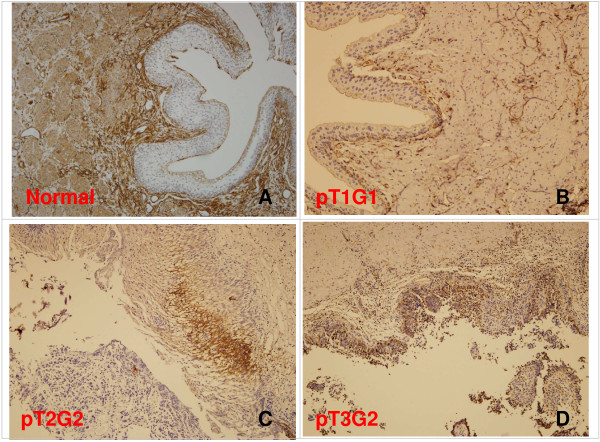
**Immunoreactivity staining of annexin A2 expression from patients, using immunohitochemistry (IHC). (A)** normal urothelium, positive in stroma, negative in urothelium. **(B)** pT1G1, negative in urothelial carcinoma. **(C)** pT2G2, positive in urothelial carcinoma **(D)** pT3G2, positive in urothelial carcinoma. Magnification: **(A,B,C,D)** × 100.

The immunoreactive staining detected by annexin A2 antibodies appeared in layers of UTUC tissue and normal tissue confined to the two proteins in the cytoplasm of the epithelium. The immunoreactive expression of the tumor tissue is stronger than that in normal tissues (Figure 6). The positive staining rate of urothelial carcinoma was 84.6% (11/13), compared to control with 7.7% (1/13). All 2 UTUCs with negative annexin A2 expression were pT1G1. The only normal urothelium with postive expression was harvested from a patient with pT3G3 (Table 3). The sensitivity and specificity of annexin A2 expression were 50% (2/4) and 100% (0/4) for pT1, 100% (4/4) and 100% (4/4) for pT2, and 100% (5/5) and 80% (1/5) for pT3, 71.4% (5/7) and 100% (7/7) for G1, 100% (3/3) and 100% (3/3) for G2, and 100% (3/3) and 66.7% (2/3) for G3, respectively (Table 4). Anyway, the study failed to calculate statistical significance due to samll sample size.

**Table 3:** The results of immunohistochemistry of annexin A2 expression in UTUC and normal tissues by patient (+: positive expression, -: no negative expression)

**ID**	**T stage**	**Tumor grade**	**UTUC**	**Control**
1	T1	G1	-	-
2	T2	G2	+	-
3	T2	G1	+	-
4	T1	G1	+	-
5	T3	G3	+	-
6	T2	G1	+	-
7	T3	G3	+	-
8	T2	G1	+	-
9	T3	G3	+	+
10	T3	G2	+	-
11	T1	G1	-	-
12	T1	G1	+	-
13	T3	G2	+	-

**Table 4:** Summary of annexin A2 expression in UTUCs and controls

**Annexin A2**	**UTUC**		**Control**	
**Expression**	**Positive**	**Negative**	**Positive**	**Negative**
T Stage				
T1 (N = 4)	2 (50%)	2 (50%)	0 (0%)	4 (100%)
T2 (N = 4)	4 (100%)	0 (0%)	0 (0%)	4 (100%)
T3 (N = 5)	5 (100%)	0 (0%)	1 (20.0%)	4 (80.0%)
Grading				
1 (N = 7)	5 (71.4%)	2 (28.6%)	0 (0%)	7 (100%)
2 (N = 3)	3 (100%)	0 (0%)	0 (0%)	3 (100%)
3 (N = 3)	3 (100%)	0 (0%)	1 (33.3%)	2 (66.7%)

## Discussion

The differential proteins in UTUC patients and those in healthy controls were compared using proteomic analysis in this study. The ages of UTUC patients and healthy controls were 62.6 years (range 33–80 years) and 34.2 years (range 18–54 years), respectively. UTUC usually occurs in older patients and elderly people appear high occurrence of progressive diseases such as type 2 diabetes mellitus and osteoarthritis. Moreover, the elderly in Taiwan often have habits of taking medicine, Chinese herbal medicine and nutritional supplements. Hence, it is clinically difficult to find healthy controls of similar age. We consider that certain urine different proteins are attributed to old age. Therefore we design an additive study to compare the proteins of normal and tumor tissues from patients with UTUC. The further study tries to clarify that those different proteins are produced by urothelial tumors rather than normal tissues.

However, non-fixed volume stepwise WAX chromatography is an effective method to fractionate urine protein. Many low-abundance proteins in the urine of UTUC patients were enriched and identified. A total of 1028 differential protein spots have been detected in the four fractions by comparative proteomic analysis. LC-MS/MS analysis has determined fifty-five protein spots. We expected some proteins expression were related to age difference. Investigation of the proteins were associated with incidence of cancers, including ZAG, CALR, annexin A2, annexin A3 and Hp [10-14]. Some of the proteins have not been reported yet in UTUC. The 2-DE data were further confirmed by western blot analysis indicating the over-expression of CALR, annexin A2, and annexin A3 in the urine and tissue of patients with UTUC in comparison with those of healthy people.

CALR, an endoplasmic reticulum (ER) chaperone, plays the role as a stress protein. The over-expression of CALR occurs in several malignancies, such as breast, prostate, liver, bladder, and lung cancers [15-18]. It has been reported that the expressions of CALR in both tumor tissue and urine of patients with bladder cancer were higher than in those of healthy people [19,20]. Elevated CALR expression was showed by 2-DE, western blot analysis, and immunohistochemistry in the urine and tissues of patients with bladder cancer [19]. Enzyme-linked immunosorbent assay was used for testing CALR with a sensitivity of 67.9% and a specificity of 80% for the detection of bladder cancer [20]. Our study for the first time showed the over-expression of CALR in the urine of patients with UTUC. Similar over-expression was also found in and the tumor tissues of patients with UTUC in comparison with healthy controls. Furthermore, in the current study this ER chaperone protein was over-expressed in the tumor areas of seven tumor/adjacent normal tissue pairs (87.5%). These data suggested that CALR is a protein secreted from UTUC tumor tissues and it is potentially a marker for UTUC.

Annexin A2 is calcium-dependent phospholipid-binding protein. It is involved in several biological processes such as immune responses, anti-inflammatory effects, Ca^2+^ transport, Ca^2+^-dependent exocytosis, and phospholipase A2 regulation. It also plays roles in the regulation of cellular growth and signal transduction pathways. Annexin A2, a potential serum marker for hepatocellular carcinoma may play an important role in liver cancer progression [21]. Down-regulation of annexin A2 in hepatocellular carcinoma cells reduced the secretion of MMP, migration ability, and invasive potential and also affected the cytoskeleton rearrangement of tumor cells [22]. Annexin A2 protein may play an important role in carcinogenesis of oral squamous cell carcinoma (OSCC). It may also serve as a potential biomarker for different pathological grade of this tumor [23]. This study is the first report on the presence of annexin A2 in the urines of patients with UTUC. The expression of annexin A2 in the tumor tissue of a patient with UTUC was higher than in the normal tissue of the same patient by western blotting analysis and immunohistochemistry and the over-expression was also found in the tumor areas of eleven tumor/adjacent normal tissue pairs (84.6%). These data in our study strongly support that annexin A2 is a crucial marker for UTUC.

Annexin A3 belongs to a family of phospholipid and calcium binding proteins. It has been implicated in cell migration and differentiation, immunomodulation, bone formation and mineralization [24]. Annexin A3 also play roles in cancer dependent autoimmne regulation against angiogenesis [25], exosomes/prosteasomes [26,27], and calcium dependent processes. Recently studies indicated annexin A3 is an inversely correlated marker for colorectal cancer [28], ovarian cancer [29], and prostate cancer [30]. It has been shown that annexin A3 in urine with a highly specific noninvasive marker for prostate cancer early detection [27,31]. Similar to annexin A2, we for the first time showed that the over-expression of annexin A3 in the urine and tumor tissues of patients with UTUC in comparison with healthy controls while annexin A3 was over-expressed in the tumor areas of eight tumor/adjacent normal tissue pairs (61.5%). Even though two UTUC tumor tissues and most normal tissues did not express annexin A3, both annexin A2 and annexin A3 showed stronger expression in UTUC urine samples than those in the urine of healthy control. This demonstrates that high-level expression of annexin A2/A3 in human urine samples still highlights their biomedical and diagnostic value for UTUC.

ZAG is a special protein. It stimulates lipid degeneration in adipocytes [32] and appears over-expressed in certain tumors and has been often recognized as a possible cancer marker [33-35]. It has been reported that ZAG initially appeared in urinary tract luminal surface. During the progression of the tumor, it transformed to the basal location. The highest level of ZAG among invasive bladder cancer sites was at the stage pT2-3 [33]. Hp promotes the accumulation of hydroxyl radicals which causes oxidative tissue damages [36]. Hp exists in two allelic forms in human body, denoted as Hp1 and Hp2. Elevated expression of Hp2 was reported in the plasma of patients with pancreatic, cervical, and breast cancer [36-38]. The results of this study demonstrated that ZAG and Hp had a stronger intensity in the urines of UTUC patients than in those of healthy people. However, higher expression of ZAG and Hp was not found in the western blot data of tumor tissues. We propose that the over-expression of ZAG and Hp in the urine of UTUC patients compared with that in healthy people, even though not consistent with the data in tumor tissues, it still somewhat reflects the possibilities of diseases or tumors in the urinary tract.

## Conclusions

Comparative proteomic analysis was conducted to investigate potential tumor markers for UTUC. A non-fixed volume stepwise elution anion exchange chromatography using DEAE-Sephacel as gel resin was employed to fractionate the total urine into four fractions prior to performing 2-DE. The 2-DE map of the urine of patients with UTUC showed a total of 1,028 proteins spots. Among them, fifty-five differential spots were identified. Three proteins, CALR, annexin A2 and annexin A3 presented with over-expression in both the urine and tissues of UTUC patients in comparison with that in the healthy and normal counterparts. The data on the urine and tumor tissues obtained from UTUC patients which were discovered and verified by proteomic and immunological analysis strongly suggested that CALR, annexin A2 and annexin A3 are essential proteins secreted from UTUC tumor tissues. Our study also established the biomedical linkage of the critical over-expression of CALR, annexin A2 and annexin A3 between the urine and tumor tissues of UTUC patients. CALR, annexin A2 and annexin A3 are very likely a panel of crucial markers in UTUC patients.

## Abbreviations

2-DE: Two dimensional gel electrophoresis; CALR: Calreticulin; Hp: Haptoglobin; UTUC: Upper tract urothelial carcinoma; WAX: Weak anion exchange; ZAG: Zinc-alpha-2-glycoprotein.

## Competing interests

The authors declare that they have no competing interests.

## Authors’ contributions

CL carried out the proteomic study, participated in the design of the study, the collection of urine and tissues, performed the 2-DE and the western blot analysis, and drafted the manuscript. JL carried out the 2-DE, western blot analysis, and immunoreactivity. HH participated in the 2-DE. YK, JH, and JC participated in the design of the study. ID participated in the design of the study and drafted the manuscript. YW conceived of the study, and participated in its design and conordination, and drafted the manuscript. All authors read and approved the final manuscript.

## Pre-publication history

The pre-publication history for this paper can be accessed here:

http://www.biomedcentral.com/1471-2407/14/363/prepub
